# Ranking Preventive Interventions from Different Policy Domains: What Are the Most Cost-Effective Ways to Improve Public Health?

**DOI:** 10.3390/ijerph17062160

**Published:** 2020-03-24

**Authors:** Nina van der Vliet, Anita W.M. Suijkerbuijk, Adriana T. de Blaeij, G. Ardine de Wit, Paul F. van Gils, Brigit A.M. Staatsen, Rob Maas, Johan J. Polder

**Affiliations:** 1 National Institute for Public Health and the Environment (RIVM), 3720 BA Bilthoven, The Netherlandsarianne.de.blaeij@rivm.nl (A.T.d.B.); ardine.de.wit@rivm.nl (G.A.d.W.); paul.van.gils@rivm.nl (P.F.v.G.); rob.maas@rivm.nl (R.M.); johan.polder@rivm.nl (J.J.P.); 2 Tilburg School of Social and Behavioral Sciences, University of Tilburg, 5000 Tilburg, The Netherlands; 3 Julius Center for Health Sciences and Primary Care, University Medical Center Utrecht, Utrecht University, 3584 CG Utrecht, The Netherlands

**Keywords:** cost-effectiveness, preventive interventions, cross-sectoral, ranking, health

## Abstract

It is widely acknowledged that in order to promote public health and prevent diseases, a wide range of scientific disciplines and sectors beyond the health sector need to be involved. Evidence-based interventions, beyond preventive health interventions targeting disease risk factors and interventions from other sectors, should be developed and implemented. Investing in these preventive health policies is challenging as budgets have to compete with other governmental expenditures. The current study aimed to identify, compare and rank cost-effective preventive interventions targeting metabolic, environmental, occupational and behavioral risk factors. To identify these interventions, a literature search was performed including original full economic evaluations of Western country interventions that had not yet been implemented in the Netherlands. Several workshops were held with experts from different disciplines. In total, 51 different interventions (including 13 cost saving interventions) were identified and ranked based on their incremental cost-effectiveness ratio (ICER) and potential averted disability-adjusted life years (DALYs), resulting in two rankings of the most cost-effective interventions and one ranking of the 13 cost saving interventions. This approach, resulting in an intersectoral ranking, can assist policy makers in implementing cost-effective preventive action that considers not only the health sector, but also other sectors.

## 1. Introduction

Globally, 48% of the disease burden is attributed to environmental, occupational, metabolic and behavioral risk factors [1]. The environments that people live in as children combined with their personal characteristics, have long-term effects on how they age [2]. Social environments also influence the development and maintenance of healthy behaviors. Maintaining healthy behaviors throughout life contributes to reducing the risk of non-communicable diseases and improving physical and mental capacity [3]. In Western Europe, the five most prevalent modifiable metabolic and behavioral risk factors for chronic disease are high Body Mass Index (BMI), high fasting plasma glucose, high systolic blood pressure, tobacco use, and alcohol use [1]. Environmental risk factors are responsible for 16% of the disease burden in the WHO European Region [4,5] and 4% of the overall disease burden in the Netherlands [6]. For instance, air pollution is the second leading cause of death from noncommunicable diseases after tobacco smoking [4].

The burden of non-communicable diseases such as cardiovascular diseases, cancer, and mental illnesses in high-income countries is expected to increase, which presents both a challenge and an opportunity for action [7]. Evidence-based action is necessary to curb these increasing trends [8]. Importantly, the literature shows that health care accounts for a mere 5% to 15% of premature mortality [9]. Health is determined by many other factors, including behavioral, social and socioeconomic factors [10,11,12]. In addition, it has been argued that health determinants should be studied taking the environmental contexts that shape them into account, especially since many health outcomes are spatially patterned [11]. Not only policies that are aimed directly at reducing risk factors for diseases or improving health, but also policies from other sectors such as agriculture, energy, and transportation may (in)directy impact health [2].

In November 2018, the Dutch Ministry of Health, Welfare and Sports presented the National Prevention Agreement (NPA) [13]. Within this NPA, interventions targeting tobacco use, obesity, and problematic alcohol use were prioritized as ways to induce lifestyle changes. However, other preventive interventions than those included in the NPA can also contribute significantly to a healthier population and can include several types of instruments, among which fiscal interventions (taxes and subsidies), regulations, changes in the built environment (infrastructure, buildings, and green space), and education and information campaigns. In the long run, interventions in health promotion and the environment can add to healthy life years and generate economic returns [14].

Investing in preventive programs, however, is challenging for policy makers, since health budgets have to compete with other major governmental expenditures. Economic evaluations offer the possibility to assess and compare costs and effects related to various preventive interventions. Moreover, they provide information about the most cost-effective (or cost saving) preventive strategy to reduce the burden of non-communicable diseases and the allocation of public resources in the most efficient way. An intervention is generally considered to be cost-effective when health gains (or averted health losses), often represented by a quality-adjusted life years (QALY) gained or a disability-adjusted life years (DALY) averted cost below a monetary reference value. When the cost of the new intervention and associated future health costs are lower than the costs resulting from current practice, an intervention is considered to be cost saving [15,16].

Others have assessed and compared interventions based on their cost-effectiveness, for example interventions that target specific health problems such as cancer [17] or non-communicable diseases [18], or more broadly evaluating preventive health interventions from a broad range of health indicators (e.g., mental health, diabetes, nutrition) [19,20,21]. In addition, the WHO has made an overview of economic evaluations of environmental health interventions and how to conduct these [22].

This study, in which we have ranked preventive interventions from different policy domains, aimed to add to existing literature by identifying and comparing cost-effective and cost saving preventive interventions from sectors other than the health sector, including interventions targeted at the physical and social environment. In particular, we assessed which preventive interventions are promising in the Dutch context by quantifying anticipated costs or savings and health effects. Ranking these interventions based on cost-effectiveness ratios provides information to decision makers on the efficiency of interventions. Results of this exploration can contribute to more integrated and effective public health policy making from an EU perspective.

## 2. Materials and Methods

To identify new promising preventive interventions, we supplemented evidence from the existing literature with input from experts working in health and environment.

### 2.1. Literature Search

A literature search was performed, using the Dutch website kosteneffectiviteitvanpreventie.nl for publications on preventive interventions to improve health from 1 January 2005 up to 1 November 2018. This website presents an overview of English language publications in PubMed regarding evidence-based economic evaluations targeted at prevention. All publications on this website were systematically assessed based on selection criteria described in Section 2.3. For search strings used in this website, please see the Appendix in Supplementary Materials. Google Scholar was used in an additional snowball method search using the publications found on this website. To identify publications on interventions that can promote health but are accomplished in the environment (for instance traffic safety, air pollution, noise nuisance), mostly grey literature was used (e.g., the INter-sectoral Health and Environment Research for InnovaTion (INHERIT) database). This database is an online resource of relevant practices in the areas of energy efficient living, green space, active travel and food consumption [23]. 

### 2.2. Expert Meetings

During the research project, we organized three group meetings with experts. We started with a kick-off workshop with experts from the different areas of the National Institute for Public Health and the Environment (RIVM) to collect information about promising interventions, which were not addressed so far in Dutch policies. At the end of our research project, we discussed our findings from the literature twice: first with experts from the RIVM and secondly with scientists from other institutes and universities, policy makers from ministries, municipal health organisations, and other relevant societal organisations. A wide range of expertise was represented in these meetings, from health and environment to behavioral sciences and health economics. After presenting our results, experts held structured small group discussions and then presented their main conclusions in a plenary session, followed by a plenary group discussion. Based on their recommendations, information from literature not yet published and scientific reports could be included. 

### 2.3. Selection Criteria

Selection criteria included original full economic evaluations of interventions from Western countries that had not yet been introduced in the Netherlands. In a full economic evaluation, two or more alternative courses of action are compared in terms of both costs and consequences. We compared interventions to usual care or doing nothing, in terms of monetary costs (€) and health consequences (DALYs). Other criteria included that the interventions had to be related to a disease with an important burden in the Netherlands, as identified and quantified by the Public Health Forecast Studies VTV2018 depicted on the Dutch website volksgezondheidenzorg.info. In addition, only English or Dutch studies from 2005 onwards were included, in which the perspective (e.g., health care) was described, and sensitivity analyses were conducted. Finally, the interventions had to be promising with respect to effectiveness and cost-effectiveness, meaning that the interventions were cost saving (when the costs of new intervention and associated future healthcare costs were lower than current practice) or had an incremental cost-effectiveness ratio (ICERs) below the Dutch cost-effectiveness reference value of €20,000 per quality-adjusted life year (QALY) gained or disability-adjusted life year (DALY) averted. Therefore, health effects had to be expressed in DALYs or QALYs [15]. All type of interventions with health effects that could be expressed in either QALYs or DALYs were eligible, including health protection and environmental interventions.

### 2.4. Cost-Effectiveness

The ICER is a metric used in cost-effectiveness analyses to summarize the cost-effectiveness of an intervention. It is defined by the difference in cost between two strategies: the costs associated with the new interventions and the costs following current practice, divided by the difference in effect in both situations. The QALY is a composite health measure combining quality of life and duration of disease, in which a QALY weight of 1.0 represents full health and 0.0 represents death. In this study, we assumed that the QALY is the complement of the DALY since the DALY is a measure of overall disease burden, expressed as the number of years lost due to illness, disability or early death [16,24]. Most of the studies on impact of environmental interventions are described in scientific reports. These reports often did not include an ICER, but for example a decrease in kg emissions or life years lost. In those cases we collected additional literature and recalculated health effects to either DALYs or QALYs ourselves. In addition, some studies used life years saved as an outcome measure. For the healthcare interventions, we recalculated the number of life years to DALYs by multiplying them with 1.12, based on Barrios et al. [25].

### 2.5. Data Extraction

We extracted information from the publications following the 24-items checklist of the Consolidated Health Economic Evaluation Reporting Standards (CHEERS) statement [26]. The principal items considered in this study were: the description and type of the intervention, health state values used, results expressed in ICERs, incremental QALYs gained or DALYs averted per patient/citizen, intervention costs and incremental costs per patient/citizen, the perspective, discount rate and time horizon used. If more studies were available addressing the same intervention, we selected the economic evaluation with the most complete information regarding the CHEERS checklist. To be able to compare costs and cost-effectiveness ratios of the economic evaluations with different base years and different currency units, all local currencies were first transferred to the Euro currency values of that time, using data on purchasing power parity of the Organization for Economic Co-operation and Development (OECD). Next, they were recalculated to 2015 € values, using the consumer price index of Statistics Netherlands. We expressed all health effects in DALYs assuming that averting a DALY was equivalent to gaining a QALY.

We estimated a conservative and maximum number of people who are eligible and expected to use the intervention, as well as the associated costs or savings and DALYs averted, called hereafter the Conservative Averted DALYs (CAD) and the Maximal Averted DALYs (MAD). We ranked the interventions based on their ICER and on their CAD and MAD, because the first describes the efficiency of the intervention in terms of costs and the latter describes the potential of the intervention in terms of reduced disease burden. Using ICER, CAD and MAD allowed us to provide complementing insights into both cost-effectiveness and actual reduced disease burden. Interventions may have a low ICER and thus rank high in the ranking based on ICER, but may reach only a limited population group, thus ranking low when based on MAD/CAD and ultimately have limited population health impact. Comparing both rankings allows policymakers to make better informed decisions. To estimate a conservative (realistic) number of participants, we divided all interventions into 6 different groups of interventions, with different coverage of the population: (1) environmental interventions: 100% coverage (e.g., improved road safety); (2) regulations: 100% coverage (e.g., reformulation of food products); (3) education and campaigns: 1% coverage (e.g., media campaign); (4) individual screening and advice: 1% participation (e.g., from a general practitioner); (5) individual support: 1% participation (e.g., individual counselling in combination with an e-health intervention); and (6) population-based screening (variable participation rates, depending on the kind of screening). MAD is determined by the total number of persons in the population concerned. The actual averted DALYs will depend on many (e.g., practical) factors. Therefore, providing both MAD and CAD estimates gives an informative range, that can sometimes be quite wide. Incremental costs and DALYs averted per person were multiplied with the conservative and maximum number of people to assess total costs and DALYs averted. Demographic figures were taken from Statistics Netherlands and were based on the year 2017.

## 3. Results

### 3.1. Included Interventions

**Figure 1:**
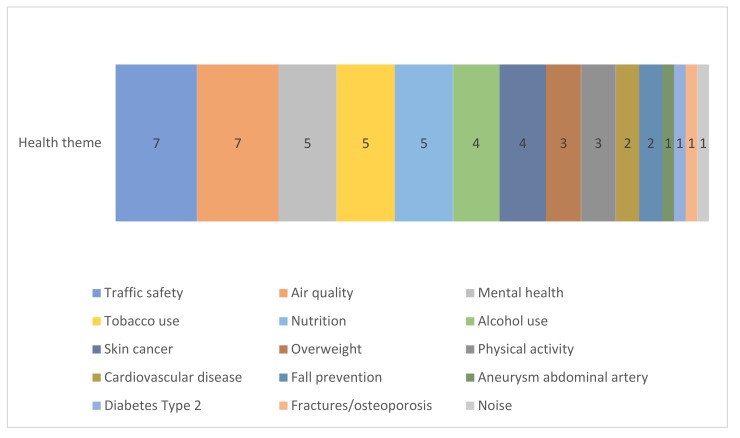
Types of health themes in the 51 interventions.

**Figure 2:**
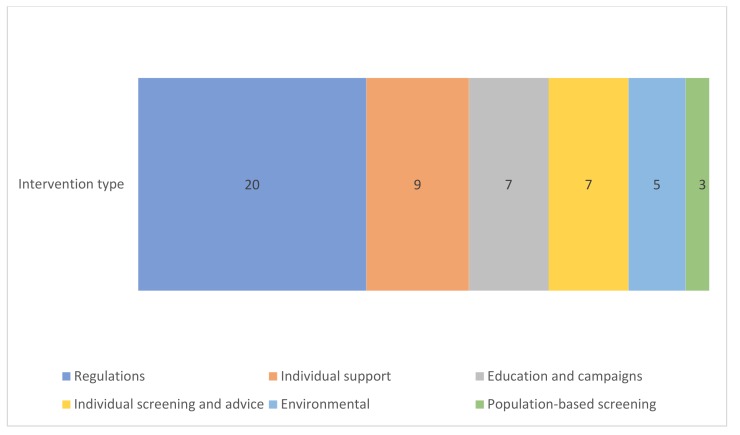
Types of interventions in the 51 interventions.

In total, 56 interventions were identified. However, as described in the Methods section, five interventions were excluded from rankings because they were different variations of the same measure, meaning that within the economic evaluation, several ICERs were given for variations of the measure (e.g., different percentages of tax increase on tobacco). In this case, the most cost-effective or the one with the highest CAD were included in rankings. Rankings were made with the remaining 51 interventions, which are described in detail in Table 1. The 51 identified interventions consist of different types of interventions (see Table 1). See Supplementary Materials (Table S1) for full details on each intervention. In Figure 1 and Figure 2, the health themes and type of interventions are presented for these 51 interventions. We did not include any occupational interventions that corresponded to our inclusion criteria. Twenty interventions involve regulations, such as a ban on tanning beds or restricting access for the most polluting cars. Among the regulations interventions are six that involve financial regulation interventions such as food taxes or a progressive fine system. Ten interventions involve individual support, such as a community exercise nutrition program for older adults, or a group based therapy for adolescents with depressed parents and who had increased depression risks themselves. Five environmental interventions were included, all directed at traffic safety, such as the installation of separate cycling paths. Three interventions involved population-based screening, e.g., for aneurysms and skin cancer. In addition, seven interventions involved individual screening and advice, which includes opportunistic screening strategies (screening in people who visit health care providers for other health reasons, often conducted by General Practitioners) or screenings that are coupled with individual advice, such as tobacco and alcohol use screenings. 

### 3.2. Cost Saving Interventions

**Figure 3:**
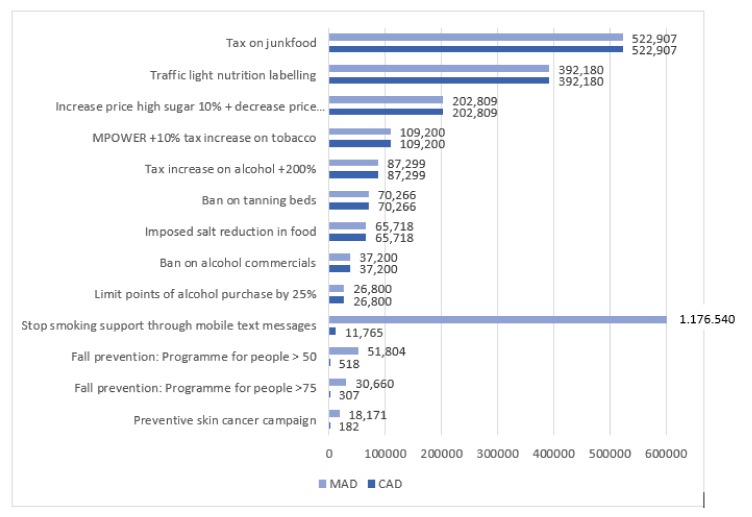
Cost saving interventions (n = 13) ranked on Conservative Averted DALYs (CAD), with Maximal Averted DALYs (MAD).

All cost saving interventions and policies in our database (13 out of 51) were ranked separately from the cost-effective interventions (37 out of 51), according to their CAD, which allowed us to see how many DALYs could realistically be averted when this intervention would be implemented (see Table 2 and Figure 3). The cost saving interventions include a range of different public health themes and intervention types (see Table 2). The ranking’s top three consisted of a ‘junk food tax’ having the highest amount of CAD (CAD = 530,573), followed by a ‘traffic light nutrition labelling intervention’ (CAD = 522,907) and another nutrition intervention, namely ‘a tax on sugar’ (CAD = 202,809). The lowest ranked cost saving intervention was a ‘preventive campaign for skin cancer’ (CAD = 182).

The CAD of the interventions presented in Table 2 is often the same as the MAD, as many cost saving interventions can realistically reach 100% of the population. This is due to the nature of these interventions: most of them are financial, regulatory, or environmental interventions affecting the whole population. However, for some interventions, CAD and MAD are different (indicated by an asterisk in the table). Their maximal reach is much higher than their realistic reach (1% of the total target population). If the cost saving interventions would be ranked based on MAD, these interventions would be ranked differently and some of these interventions (four out of 13 interventions, such as fall prevention program or a skin cancer preventive campaign) would be higher in ranking. 

### 3.3. Cost-Effective Interventions

**Figure 4:**
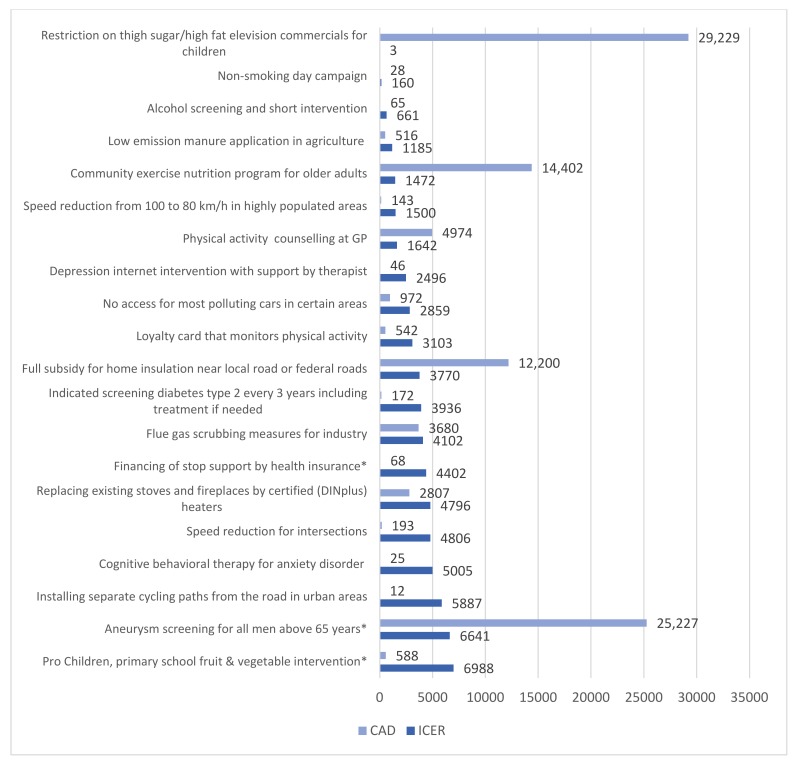
Top 20 ranking most cost-effective interventions (based on ICER € 2015), with Conservative Averted DALYs (CAD).

Out of the 37 cost-effective interventions, we constructed a top 20 of most cost-effective interventions and policies, based on their ICER. This ranking is presented in Table 3 and in Figure 4, the CAD is also presented. Table 3 also presents the types of interventions and health themes of the top 20 most cost-effective interventions, and shows that eight interventions concerned regulation interventions; four were individual support interventions and three were education and campaigns interventions. In addition, three individual screening and support interventions were included, and for both environmental and population-based screening, one cost-effective intervention was identified. There were different health themes among the top 20 most cost-effective interventions. The largest theme group included eight lifestyle-related interventions, followed by five air quality interventions, two chronic disease interventions, two traffic safety interventions, two mental health interventions and a noise intervention. 

The interventions and policies ranged from an ICER of 3 to an ICER of 6988, with a ‘restriction on television commercials on high fat/high sugar foods and beverages for children’ having the lowest ICER of 3, and ‘Pro Children, primary school fruit & vegetable intervention’ having the highest ICER of 6988.

Several interventions, indicated by an asterisk, have different CAD and MAD, because their maximum coverage is much higher than the estimated conservative coverage (for most, this was set at 1% as described in the Methods section).

### 3.4. Averted DALYs

**Figure 5:**
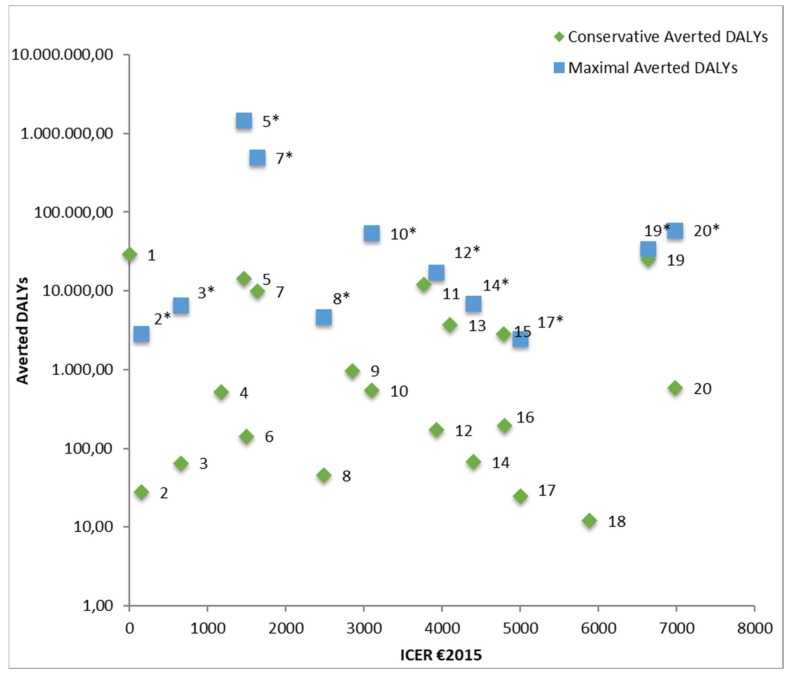
ICER and CAD of the top 20 interventions with log transformation for y-axis (labels correspond to intervention labels in Table 2). * Interventions marked by an asterisk (*) have different CAD and MAD, because the total number of persons in the population concerned is much higher than their conservative reach (1% of the total target population). 5* (MAD = 1,440,174), 7* (MAD = 497,365) and 10* (MAD = 54,180) have a markedly higher MAD than the others interventions.

The most cost-effective interventions (based on ICER) do not necessarily have the highest amount of conservative averted DALYs. In order to visualize which interventions are both highly cost-effective and result in the most conservative averted DALYs, Figure 5 is presented, in which the € 2015 ICER and CAD are plotted against each other. Data labels for each intervention correspond to data labels in Table 3. 

As can be seen in Figure 5, there are several interventions that have both a low ICER and a relatively high CAD (1 = ‘restriction on television commercials with high sugar/high fat foods and beverages for children’, 5 = ‘community exercise nutrition program for older adults’, 7= ‘physical activity counselling at a GP’). These interventions are thus very cost-effective and have a high DALY impact at the same time. In addition, still with relatively low ICERs and high CAD are 11 (‘full subsidy for home insulation near local road or federal roads’), 13 (‘flue gas scrubbing measures for industry’), 15 (‘replacing existing stoves and fireplaces by certified (DINplus) heaters’), and 19 (‘aneurysm screening for all men above 65 years’). There are several interventions that have a favorable ICER but that also result in relatively low conservative averted DALYs (CAD <100) if implemented. These include 3 (‘screening and short intervention for alcohol use’), 8 (‘depression internet intervention combined with therapist support’), 18 (‘installing separate cycling paths from the road in urban areas’).

From the top 20 cost-effective interventions, 11 have different CAD and MAD, because the total number of persons in the population concerned is much higher than their conservative reach (1% of the total target population). If the interventions would be ranked based on MAD, these 11 interventions would be ranked differently, most would be higher in ranking. Some interventions would increase by thousands of DALYs averted (such as ‘depression internet intervention’, from CAD = 46 to MAD = 4636 and ‘non-smoking day campaign’ from CAD= 28 to MAD = 2820), but there are some interventions that have a substantial difference of thousands of DALYs averted (e.g., ‘community exercise nutrition program for older adults’ from CAD = 1472 to MAD = 14,402 and ‘physical activity counselling at GP’ from CAD = 4974 to MAD = 497,364).

### 3.5. Ranking Interventions on a Conservative Estimate of DALYs Averted

In order to get an insight into which interventions can have the largest impact on DALYs averted, we also ranked all 51 interventions based on their CAD, which resulted in a different top 20 than the one ranked based on the ICER (see Table 4). It is important to note that most ICERs in this CAD-based top 20 are still considerably lower than the (low) Dutch threshold for cost-effectiveness, i.e., 20,000 € per QALY for diseases with the lowest disease impact [15].

The CAD top 20 contains many interventions that are also cost saving (n = 10), with nutrition interventions ranking particularly high. These cost saving interventions appear to have a relatively large impact on averted DALYs, in the most cost-effective way. As can be seen, many of these interventions include financial or regulatory interventions that can affect a great part of the population. Of cost-effective interventions included in this top 20, several stand out that have a high CAD and a low ICER, among which are a ‘restriction on television commercials with high sugar/high fat foods and beverages for children’, and an ‘aneurysm screening for all men above 65 years old’.

### 3.6. Comparison of the Top 20 Rankings Based on ICER and CAD

Fifteen interventions that were in the top 20 based on ICER, did not make the top 20 based on CAD, including some that were ranked very high in the top 20 based on ICER, such as ‘non-smoking day campaign’, ‘alcohol screening and short intervention’, and ‘Low emission manure application in agriculture’. Thus, although these interventions are highly cost-effective, they have a relatively lower impact on conservative averted DALYs. 

In addition, ten interventions in the top 20 based on CAD were cost saving and not included in the top 20 based on ICER. The remaining ten interventions in the top 20 based on CAD consisted of five new interventions that were not included in the cost-effectiveness top 20 nor were cost saving, namely ‘screening vitamin D deficiency and supplementing (>65 years)’, ‘introducing a progressive fine system’, ‘a tailored lifestyle intervention for persons with BMI>25, aged 30-75’, ‘creating non-crossable central reservation on roads’ and the ‘polypill (without aspirin) for 7.5% risk population’. Although these interventions have relatively higher ICERs, the ICERs are still acceptable according to Dutch standards, and implementing these interventions can result in a relatively high number of averted DALYs.

Five other interventions were included in both the top 20 of most cost-effective interventions and the top 20 highest of CAD interventions. They were all in different positions in the rankings, with only ‘aneurysm screening for men aged over 65’ ranked higher in the top 20 based on CAD compared to the top 20 based on ICER. The others all ranked lower in the top 20 based on CAD, including ‘community exercise nutrition program for older adults’, ‘physical activity counselling at general practitioner’, ‘restrictions on television commercials with high fat/high sugar foods and beverages’, and ‘full subsidizing of home isolation for noise near local roads’. 

## 4. Discussion

The aim of this study was to give policy makers new ideas for health prevention and promotion policies, combining different sectors including the health, transport, food and environmental sectors. In this study, we identified cost-effective preventive interventions that may result in an increased healthy life expectancy in the Netherlands. These interventions are related to different policy perspectives and can be implemented in different sectors. We assessed and ranked interventions targeting metabolic, environmental, occupational and behavioral risk factors. In this study, we identified more than 50 examples of cost-effective interventions, including 13 cost saving interventions. We ranked these cost-effective interventions, resulting in a top 20 of most cost-effective interventions based on ICER and a top 20 of most cost-effective interventions based on Conservative Averted DALYs (CAD). In addition, we ranked the 13 cost saving interventions on CAD. The results of this study can contribute to the implementation of cost-effective policies, and a more optimal distribution of scarce resources.

The top three cost saving interventions consists of a junk food tax, a traffic light nutrition labelling intervention, and a sugar tax. The top three cost-effective interventions (based on ICER € 2015) consists of a restriction on television commercials with high sugar/high fat foods and beverages for children, a non-smoking day campaign, and a screening and short intervention targeted at alcohol use. Despite the intersectoral perspective taken, it is remarkable that the six highest ranking cost-effective interventions all target classical risk factors, including tobacco, alcohol, and nutrition.

In the Netherlands, a preventive health intervention is seen as cost-effective if the ICER is below 20,000 euro per Quality Adjusted Life Year [64]. Due to different societal and political preferences, different thresholds may be set for preventive policy interventions taken in different policy sectors and in other countries [65], for example due to context impacts. Knowing that the reference value for the cost per QALY in environmental social cost benefit analyses in the Netherlands is between € 50,000 and € 100,000 [66], we chose a conservative threshold and looked only at the lower value currently used in preventive health policy.

We assessed these preventive interventions simultaneously by quantifying incremental costs or savings, health effects, and the cost-effectiveness ratio. Cost-effectiveness is one of the arguments to decide whether or not to implement an intervention. This assessment can be used as a starting point for thinking on how to allocate resources to preventive policies aimed at increased healthy life expectancy. 

Cost-effectiveness analyses may help to gain insight into which policies have the best balance between costs and health effects. In addition to the efficiency argument, other arguments are relevant though, such as other societal impacts (e.g., biodiversity), equity, individual liberty, joy, public awareness and support, ethical, and budget arguments. An example of a Dutch policy-decision based on other arguments than cost-effectiveness is aneurysm screening. This intervention is assessed as a cost-effective intervention (see Table 1). Nevertheless, the Health Council of the Netherlands advised to not introduce such screening because the incidence of aneurysms in the Netherlands is currently declining [67].

Different political decision makers are financially responsible for specific preventive interventions. This makes it important to consider differences in perspectives between stakeholders who are confronted with the net payments for implementing policies and stakeholders who experience the financial gains from these policies. The decision regarding which preventive interventions to invest in will depend on the financial resources that are available to allocate by the decision maker and the costs and benefits of alternative uses of that budget. Implementation of a cost-effective intervention does not necessarily imply that overall healthcare costs will be reduced. On the contrary, as only new interventions are included in this analysis, investment costs have to be considered. Even an overall cost saving intervention needs investments. It might be that budget constraints make it impossible to choose the most cost-effective intervention or to select only a few interventions from the large amount of interventions. In parallel, investments made for other reasons, for example green space needed for climate adaptation, can also be beneficial for health. A subsequent step is to consider whether substitution of existing interventions by more cost-effective interventions is feasible and acceptable. For example, a tax intervention implies low investment costs, but this intervention can raise issues of equity as imposing taxes would impose a heavier burden on low-income households than on high-income households. This argument that is it unfair to tax unhealthy food could be contested when not only the monetary costs but also the beneficial health effects of taxation are taken into account [68]. A systematic review on health taxes concluded not only that there is evidence for positive impacts of high tax rates on health behaviors and outcomes, but also that these health outcomes are likely to be largest for lower income groups [69]. A possible widening of inequality in the income dimension may thus be counteracted by reduced inequality in the health dimension. 

The discussion of the comparability of the ICER estimates included in this analysis, focused on the assumptions, data and calculations underlying the ICER estimates. For each health intervention, we collected the most complete information relevant for the economic evaluation, based on the CHEERS checklist. For the environmental interventions, we had to combine different sources (mostly scientific reports) and had to made assumption to calculate an ICER value. An important assumption is for instance that one QALY gained equals one DALY averted. Due to these calculations and assumptions we were able to present the order of magnitude of the ICER value of the different interventions. We assessed the ICER estimates as presented in the studies. We included time horizon of the health impact and discount rate in the database. The time horizon of the interventions differs between one year and life time. As different discount rates are used in the different studies describing the interventions, this might influence the rating of the intervention. 

As a consequence of the underlying assumptions, it is impossible to make definitive conclusions about the preferred order of the preventive interventions based on the costs effectiveness estimates. 

To estimate the conservative averted DALYs (CAD), based on the maximal averted DALYs (MAD), we assumed a cautious 1% participation rate for screening and advice interventions, and for individual support interventions. Probably, this is an underestimation of the cost-effectiveness of these interventions. 

The included environmental interventions are traffic safety, air quality and noise reduction interventions. In this analysis, most of the ICER values of the traffic related interventions are based on statistical estimates and not on empirical experiments with real life patients or victims. For air pollution, the association between air pollution levels and health impacts, derived from epidemiological studies, is the basis for the health impact analysis. The health impact of air quality interventions is based on the assumption that the whole population will experience a lower risk. The health impact of a full subsidy on noise reduction interventions of highly exposed houses is estimated under the assumption that all people living in a house with a noise exposure above the standard of 65db, have a health risk due to noise and that this risk is fully eliminated after taking the intervention. Not all the costs of interventions have been monetized and included in the ICER. This includes for example the welfare loss due to a ban on using fireplaces, or the extra travel time due to speed limits. Moreover, the costs of the environmental interventions do not include the costs of enforcement. However, costs could also be an overestimation as learning effects or economies of scale have not been assumed. Also, the impacts of climate change and energy policies is not included, as at the moment, not enough is known to quantify the health impacts of potential interventions [70]. To be able to calculate the health gains of airplane noise reduction, we used the modelled health gain given in Jiao et al. [71]. Regarding the environmental interventions, and in particular regarding noise reduction interventions, much more empirical research to estimate the health effects of a policy intervention is needed, as it is of high public interest. 

Within the scope of this project, we choose to make use of expert sessions to investigate possible cost-effective interventions. An improvement for future similar investigations would be to conduct a Delphi study to identify the most promising preventive interventions by soliciting more and a broader group of qualified experts from different relevant scientific disciplines. In addition, the interventions included in this paper are not an exhaustive list: most of our included studies were found using Pubmed, but there are other databases. In addition, studies may have been published after our search which were not included in this article, or included interventions may have been implemented in the Netherlands, rendering them no longer ‘new’ in the Dutch context.

## 5. Conclusions

We believe this is an informative approach to raise the awareness of policy makers to not only use results of studies from the health domain for public health policy making, but also use results of studies from other policy domains. Varied options to improve the health of the (Dutch) population were identified in our study. In sum, our findings provide information that is valuable for future public health policies. Society has to make difficult trade-offs between different policy options, as budgets are limited by definition. Making use of cost-effectiveness ratios and estimates of averted DALYs after introduction of policies could be helpful in making this kind of trade-offs. Improving public health needs to be done intersectorally, involving interdisciplinary cooperation between policy domains to allow for the most efficient and cost-effective approach. Next steps include considering whether substitution of existing interventions by more cost-effective ones is feasible, acceptable and can be done in an inclusive way. In this study, the approach is applied for the Netherlands, but we postulate that this is a relevant approach for all countries who want to improve the health status of their population in a cost-effective way. 

## Figures and Tables

**Table 1:** Description of all 51 cost-effective and cost saving interventions.

Theme	Intervention	Type of Intervention	Description
Aneurysm abdominal artery	Screening all men above 65 years	Population based screening	Screening for aneurysm abdominal artery with ultrasonography for all men above 65 years [27]
Air quality	Speed reduction from 100 to 80 km/h in highly populated areas	Regulations	Reduction in the speed limit on urban motorways from 100 km/h to 80 km/h [28,29,30]
Air quality	Low emission manure application in agriculture	Regulations	Tightening the low-emission fertilization on arable land, which entails that slurry on arable land can no longer be distributed above ground. The use of a drag foot is also no longer permitted. The manure must be immediately put into the soil with an injector or sod [28]
Air quality	No access for most polluting cars in certain areas	Regulations	Low emission zones tackling the more polluting heavy goods vehicles [30]
Air quality	Flue gas scrubbing measures for industry	Regulations	More efficient flue gas desulphurization plants in refining, steel and soot production and other industries [28]
Air quality	NO_2_ reduction inland navigation (selective catalytic reduction)	Regulations	A subsidy of 80% for the purchase of soot filters and SCR* systems on existing inland vessels [28]
Air quality	Replacing existing stoves and fireplaces by certified (DINplus**) heaters	Regulations	Replacing (phasing out) existing heaters and fireplaces by DINplus certified heaters. These are heaters that meet strict emission standards [28]
Air quality	Installing separate cycling paths (routes) from the main road in urban areas	Environmental	Installing extra separate cycling paths from the main road in urban areas so that cyclers can make use of these roads instead of on-road cycling [30]
Alcohol use	Ban on alcohol commercials	Regulations	A national ban on alcohol commercials (media, sponsoring, internet, product placement, direct mail and price promotions) [31]
Alcohol use	Limit points of purchase by 25%	Regulations	Decreasing the number of points of purchase of alcohol by 25% (modeled by decreasing sales concentration by 25%) [31]
Alcohol use	Tax increase +200%	Regulations (financial)	An excise tax increase of 200% of alcoholic consumptions (the excise part of the total price of alcoholic consumption is increased by 200%) [31]
Alcohol use	Screening and short intervention	Individual screening and advice	Opportunistic screening, preventing alcohol misuse by brief (15 min) consultations, providing information and support, conducted by trained staff over the phone [32]
Cardiovascular disease	Imposed salt reduction in food	Regulations	Legislation and enforcement to make ‘Tick’ salt limits mandatory for food manufacturers (Tick is an Australian program to encourage voluntary salt reduction in products) [33]
Cardiovascular disease	Polypill for those at risk	Individual screening and advice	Opportunistic screening (offered to eligible patients by general practitioners (GPs) during routine visits), if above threshold risks levels for cardiovascular mortality, lifelong preventive medication is offered [34]
Diabetes Type 2	Diabetes type 2: Indicated screening every 3 years including treatment when needed	Individual screening and advice	3-yearly risk stratified screening for the 40–74 age group. Screening at GP for high-risk of diabetes with a questionnaire, followed by standard diabetes test for high-risk group [35]
Fall prevention	Fall prevention: Program for elder people (>50)	Education and campaigns	“Healthy Steps for Older Adults” (HSOA) includes physical performance assessments of balance and mobility conducted by staff or trained volunteers, referrals for physician care and home safety if needed, a 2-hour falls prevention class involving recognition of home hazards and falls risk situations, demonstrations of exercises to improve balance and mobility [36,37]
Fall prevention	Fall prevention: Program for elder people (>75) through home care	Individual screening and advice	A home-based exercise program based on the Senior Step intervention, with self-tests, instruction books with exercises [38]
Fractures / osteoporosis	Screening vitamin D deficiency and supplementing (>65 years)	Individual screening and advice	Population screening for vitamin D insufficiency followed by treatment based on the vitamin D serum level (a ‘screen and treat’ strategy) [39]
Mental health	Cognitive behavioral therapy for anxiety disorders through internet with supportive coaching (>60 years)	Individual support	Managing Stress and Anxiety Course for adults aged 60 years, who were experiencing symptoms of stress, anxiety, and worry. The course is a five-lesson program and is delivered over 8 weeks with regular support from a clinical psychologist via a secure email system and telephone [40]
Mental health	Group-based therapy for adolescents with depressed parents and who had increased depression risks themselves	Individual support	The depression intervention consisted of 15 one-hour cognitive behavioral therapy (CBT) sessions for groups of 6 to 10 adolescents [41]
Mental health	Combination of internet intervention, supported by therapist for people 60 years and older	Individual support	Managing your mood course, 8-week treatment, cognitive-behavioral therapy with online lessons and homework [40]
Mental health	Screening and treatment of cancer patients	Individual support	Identification of major depression using a two-stage screening system in specialist cancer clinics and treatment of major depression using DCPC***: a multicomponent, systematic, team-delivered treatment program integrated with the patient’s cancer care [42]
Mental health	Intervention for informal caregiver of relatives with dementia (group / individual support) for stress and burn out problems	Individual support	A manual based coping intervention consisting of 8 individual therapy sessions for family carers of people with dementia, delivered by psychology graduates with intervention training. Carers received a CD with manual and relaxation exercises to practice at home [43]
Noise	Full subsidy for home insulation near local road or federal roads	Regulations	Full subsidy for sound insulation of residential homes near local road or federal roads for residence exposed above the limit of sound of 65 bb [44]
Nutrition	Tax on junk food	Regulations (financial)	A tax on unhealthy foods (biscuits, cakes, pastries, pies, snack foods, confectionary and soft drinks) that would raise consumer-end prices of these products by 10% [45]
Nutrition	Traffic light nutrient labelling	Regulations	A mandatory inclusion of front-of-pack traffic light labelling, coupled with a 1-year national social marketing campaign to educate and inform the population on label interpretation [45]
Nutrition	Increasing price of high sugar products with 10%	Regulations (financial)	Increasing price of high sugar products with 10% tax increase [46]
Nutrition	Restriction on television commercials with high sugar/high fat foods and beverages for children	Regulations	Banning television (TV) advertisements for energy-dense, nutrient-poor food and beverages and fast food outlets, during children’s peak viewing times [47]
Nutrition	Primary school fruit and vegetable intervention	Education and campaigns	Pro Children intervention with classroom, school, family and one optional component, including classroom curriculum with activities regarding fruit and vegetables, provision of fruit and vegetables for free, by subscription or as part of school meals [48]
Overweight	Community exercise nutrition program for older adults	Individual support	Texercise Select is a health promotion and wellness program. A 12-week program (2-week recruitment, 10-week interactive classes with physical activity, diet education, interactive group discussions) to improve knowledge, confidence, mobility, ease and fall-prevention [49]
Overweight	Tailored lifestyle intervention for persons with BMI>25, aged 30–75	Individual support	The ‘Beweegkuur’ is a combined lifestyle intervention targeting physical activity, diet and behavior [50]
Overweight	Loyalty card that monitors activity, collects points and rewards	Education and campaigns	The Physical Activity Loyalty (PAL) card scheme entails that employees from a workplace setting get a loyalty card to monitor their physical activity levels (by swiping their card at receivers placed along designated walking routes, within the grounds of their workplace), with real-time feedback. For the incentive group minutes of physical activity were also converted into points and these points could be redeemed for rewards sponsored by local businesses [51]
Physical activity	Physical activity intervention in print (instead of web-based) (>50 years)	Education and campaigns	A print-based physical activity intervention entailing tailored advice three times (in four months), targeting the psychosocial determinants of physical activity. Including comparison to others, physical activity, model stories, information on consequences of inactivity and suggestions on how to deal with barriers [52]
Physical activity	Physical activity: Pedometer linked to general practitioner (GP) visit. After identifying too little activity: providing activity advice	Individual support	GPs offer patients identified as sedentary (by a questionnaire) to use pedometers. The patients attend 3 follow-up sessions with the GP’s assistant to complete the pedometer intervention [53]
Physical activity	Physical activity counselling at GP	Individual support	Green Prescription program involving written physical activity advice developed together with the patient in a GP setting (identified as sedentary through screening by GP or practice nurse) and subsequent tailored individual advice and follow-up telephone support by exercise specialist for three months [54]
Skin cancer	Ban on tanning beds	Regulations	A national ban on (public) sunbed use [55]
Skin cancer	Lesion-directed screening	Population based screening	Invitation to get free skin cancer check of specific lesion meeting certain criteria. Screening performed by dermatologists (including examinations, treatment, and follow-up) [56]
Skin cancer	Total body examination	Population based screening	Total body examination screening. Personal invitation, with screening performed by dermatologists (consequent examinations, treatment, and follow-up if needed) [56]
Skin cancer	Preventive campaign	Education and campaigns	A sensitization, public education, comprehensive campaign on skin cancer [55]
Tobacco use	MPOWER +10% tax increase	Regulations (and financial regulations)	MPOWER**** consists of a package of measures, defined by the WHO (smoking bans, quit smoking aids, mass media campaigns, advertisements bans) and an annual excise tax increase of 10% [57]
Tobacco use	Stop smoking support through mobile text messages	Individual screening and advice	Txt2stop is a personalized smoking cessation advice and support by regular mobile phone messages (with quitting advice, distraction, support) around a quit date set within 30 days of starting the program. Also quit buddies, text service when cravings and quizzes) [58]
Tobacco use	Financing of stop support by health insurance	Individual screening and advice	The reimbursement of an integrated smoking cessation program, consisting of a combination of behavioral counselling and pharmacotherapy [59]
Tobacco use	Mass media campaign	Education and campaigns	Mass media tobacco campaign: dissemination of information through television, radio, print media and billboards, with the intention of encouraging smokers to quit, and of maintaining abstinence in non-smokers [57]
Tobacco use	Non-smoking day campaign	Education and campaigns	No Smoking Day consist of a national social marketing campaign and provides materials such as posters and leaflets to local organizations to use in events and promotional activities, to ‘help smokers who want to stop smoking by creating a supportive environment and highlighting the help available for smokers who want to stop’ [60]
Traffic safety	Creating non-crossable central reservations on roads	Environmental	Creating non-crossable central reservations on roads that separates two roads in opposite direction and prevents frontal car accidents and passing cars on all national roads [61]
Traffic safety	Introducing a progressive fine system	Regulations (financial)	A progressive penalty system in which the fine increases in the case of repeat offences (fines are currently license plate-based) [62]
Traffic safety	Vehicle technology with contour marketing on all vehicles	Regulations	Accelerated introduction of retro reflecting contour marking on all (new and old) lorries and trailers above a certain weight [63]
Traffic safety	Creating hard to cross central reservation on roads	Environmental	Creating difficult to cross central reservation on roads that separates two roads in opposite direction on all national roads [61]
Traffic safety	Roundabouts	Environmental	Reconstructing of crossovers with traffic lights or crossovers with priority arrangements to roundabouts on all national roads [61]
Traffic safety	Speed reduction for intersections	Regulations	A speed reduction for all intersections with a speed above 70k km/h and distributor roads on all national roads [61]
Traffic safety	Targeting unsafe arches, signs and compensating measures	Environmental	Measures targeting unsafe traffic arches, such as signs, reflectors and lightening on all national roads in the Netherlands were this is the best option from a road safety perspective [61]

* DINplus heaters are heaters that meet strict emission standards including usage of clean, untreated wood with high efficiency; ** SCR systems are selective catalytic reduction systems, where nitrogen oxides are converted into diatomic nitrogen and water, helping to reduce emissiions of nitrogen oxide and particulate matter; *** DCPC stands for Depression Care for People with Cancer; **** MPOWER stands for different types of measures, namely Monitoring, Protect, Offer help, Warn, Enforce bans and Raise taxes.

**Table 2:** Ranking of 13 cost saving interventions, based on conservative averted DALYs (CAD) (ranked from highest CAD to lowest CAD).

Intervention	Theme	Type of Interventions	Conservative Averted DALYs	Maximal Averted DALYs
Tax on junk food	Nutrition	Regulations (financial)	522,907	522,907
Traffic light nutrient labelling	Nutrition	Regulations	392,180	392,180
Increasing price of high sugar products with 10%	Nutrition	Regulations (financial)	202,809	202,809
Tax increase on alcohol +200%	Alcohol use	Regulations (financial)	109,200	109,200
MPOWER +10% tax increase on tobacco	Tobacco use	Regulations (and Regulations financial)	87,299	87,299
Ban on tanning beds	Skin cancer	Regulations	men: 31,440; women: 38,826	men: 31,440; women: 38,826
Imposed salt reduction in food	Cardiovascular disease	Regulations	65,718	65,718
Ban on alcohol commercials	Alcohol use	Regulations	37,200	37,200
Limit points of alcohol purchase by 25%	Alcohol use	Regulations	26,800	26,800
Stop smoking support through mobile text messages *	Tobacco use	Individual screening and advice	11,765	1,176,540 *
Fall prevention: Program for elder people (>50) *	Fall prevention	Education and campaigns	518	51,804 *
Fall prevention: Program for elder people (>75) through home care *	Fall prevention	Individual screening and advice	307	30,660*
Preventive skin cancer campaign *	Skin cancer	Education and campaigns	182	18,171 *

* These interventions have different CAD and MAD, because the total number of persons in the population concerned is much higher than their conservative reach (1% of the total target population). If the cost saving interventions would be ranked based on MAD, these interventions would be ranked differently, with most interventions ranking higher.

**Table 3:** Top 20 of cost-effective interventions (ranked from lowest to highest ICER € 2015).

Intervention	Theme	Type of Intervention	Intervention Label Figure 5	ICER
Restriction on television commercials with high sugar/high fat foods and beverages for children	Nutrition	Regulation and enforcement	1	3
Non-smoking day campaign *	Tobacco use	Education and campaigns	2	160
Screening and short intervention *	Alcohol use	Screening and advice	3	661
Low emission manure application in agriculture	Air quality	Regulation and enforcement	4	1185
Community exercise nutrition program for older adults *	Physical activity	Individual support	5	1472
Speed reduction from 100 to 80 km/h in highly populated areas	Air quality	Regulation and enforcement	6	1500
Physical activity counselling at General Practitioner *	Physical activity	Individual support	7	1642
Depression: Combination of internet intervention, supported by therapist (<60 with depression complaints) *	Mental health	Individual support	8	2496
No access for most polluting cars in certain areas	Air quality	Regulation and enforcement	9	2859
Loyalty card that monitors physical activity, collects points and rewards*	Physical activity	Education and campaigns	10	3103
Full subsidy for home insulation near local road or federal roads	Noise	Regulation and enforcement	11	3770
Indicated screening diabetes type 2 every 3 years including treatment if needed *	Diabetes Type 2	Screening and advice	12	3936
Flue gas scrubbing interventions for industry	Air quality	Regulation and enforcement	13	4102
Financing of stop support by health insurance *	Tobacco use	Screening and advice	14	4402
Replacing existing stoves and fireplaces by certified (DINplus stricter emission reducing criteria) heaters	Air quality	Regulation and enforcement	15	4796
Speed reduction for intersections	Traffic safety	Regulation and enforcement	16	4806
Cognitive behavioral therapy for anxiety disorder through internet with supportive coaching (>60 years) *	Mental health	Individual support	17	5005
Installing separate cycling paths from the road in urban areas	Traffic safety	Regulation and enforcement	18	5887
Aneurysm screening for all men above 65 years *	Cardiovascular disease	Population based screening	19	6641
Pro Children, primary school fruit & vegetable intervention *	Nutrition	Education and campaigns	20	6988

* These interventions have different CAD and MAD, because the total number of persons in the population concerned is much higher than their conservative reach (1% of the total target population).

**Table 4:** Ranking of the top 20 highest Conservative Averted DALYs (CAD), ranked from highest CAD to lower CAD (from the total of 51 interventions).

Interventions	Theme	Intervention Type	Conservative DALYs Averted	ICER 2015
Tax on junk food	Nutrition	Regulations: financial	522,907	cost saving
Traffic light nutrient labelling	Nutrition	Regulations	392,180	cost saving
Polypill (without aspirin) for 7.5% risk population	Cardiovascular disease	Screening and advice	296,000	9523
Increasing price of high sugar products with 10% tax increase	Nutrition	Regulations: financial	202,809	cost saving
Tax increase on alcohol +200%	Alcohol	Regulations: financial	109,200	cost saving
MPOWER* +10% tax increase on tobacco	Tobacco use	Regulations: financial	87,299	cost saving
Ban on tanning beds	Skin cancer	Regulations	3,882,631,440	cost saving
Imposed salt reduction in food	Cardiovascular disease	Regulations	65,718	cost saving
Screening vitamin D deficiency and supplementing (>65 years)	Fractures/Osteoporosis	Screening and advice	46,280	9559
Ban on alcohol commercials	Alcohol	Regulations	37,200	cost saving
Restriction on television commercials with high sugar/high fat foods and beverages for children	Nutrition	Regulations	29,229	3
Limit alcohol points of purchase by 25%	Alcohol	Regulations	26,800	cost saving
Aneurysm screening all men above 65 years	Aneurysm	Population based screening	25,277	6641
Community exercise nutrition program for older adults	Physical activity	Individual support	14,402	1472
Full subsidy for home insulation near local road or federal roads	Noise	Regulations: financial	12,200	3770
Stop smoking support through mobile text messages	Tobacco use	Screening and advice	11,765	cost saving
Creating non-crossable central reservations on roads	Traffic safety	Environmental	9964	7603
Introducing a progressive fine system	Traffic safety	Regulations: financial	5850	7413
Tailored lifestyle intervention for persons with BMI>25, aged 30–75	Overweight	Individual support	5640	2808–3276
Counselling for physical activity at GP	Physical activity	Individual support	4974	1642

* MPOWER stands for different types of measures, namely Monitoring, Protect, Offer help, Warn, Enforce bans and Raise taxes.

## Supplementary Materials

The following are available online at https://www.mdpi.com/1660-4601/17/6/2160/s1, Excel Table S1: Description and data on all interventions and Appendix A1 for search strings used in the literature search.
