# Supplementary material for: Ranking Preventive Interventions from Different Policy Domains: What Are the Most Cost-Effective Ways to Improve Public Health?
Source: Int J Environ Res Public Health. 2020 Mar 24;17(6):2160. doi: 10.3390/ijerph17062160 (PMC7142580; doi:10.3390/ijerph17062160)
Supplement: Supplementary file 1 [file ijerph-17-02160-s001.zip › ijerph-719031 supplementary files appendix.docx]

Supplementary files

Appendix A1: Search strings used for the intervention database kosteneffectiviteitvanpreventie.nl

("Costs and Cost Analysis"[mh] OR "Cost-Benefit Analysis"[mh] OR cost benefit*[ti] OR cost effect*[ti] OR cost utility[ti] OR cost efficien*[ti] OR econom*[ti] OR pharmacoeconomic*[ti] OR pharmaco-economic*[ti] OR (cost*[ti] AND (effect*[ti] OR benefit*[ti] OR quality[ti] OR efficien*[ti])) OR (cost*[ti] AND economics[majr])) AND (prevention[tiab] OR preventive[tiab] OR screening[tiab] OR vaccinat*[tiab] OR immunization*[tiab] OR immunisation*[tiab] OR health promotion[tiab] OR health protection[tiab] OR intervention*[tiab] OR health protection[tiab] OR health promotion[mh] OR healthy people programs[mh] OR preventive health services[mh] OR immunization[mh] OR vaccination[mh] OR health surveys[mh] OR mass screening[mh] OR life style[tiab] OR lifestyle[tiab] OR life style[mh:noexp])

("Costs and Cost Analysis"[mh] OR

"Cost-Benefit Analysis"[mh] OR

cost benefit*[ti] OR

cost effect*[ti] OR

cost utility[ti] OR

cost efficien*[ti] OR

econom*[ti] OR

pharmacoeconomic*[ti] OR

pharmaco-economic*[ti] OR

(cost*[ti] AND (effect*[ti] OR

benefit*[ti] OR

quality[ti] OR

efficien*[ti])) OR

(cost*[ti] AND economics[majr]))

AND

(prevention[tiab] OR

preventive[tiab] OR

screening[tiab] OR

vaccinat*[tiab] OR

immunization*[tiab] OR

immunisation*[tiab] OR

health promotion[tiab] OR

health protection[tiab] OR

intervention*[tiab] OR

health protection[tiab] OR

health promotion[mh] OR

healthy people programs[mh] OR

preventive health services[mh] OR

immunization[mh] OR

vaccination[mh] OR

health surveys[mh] OR

mass screening[mh] OR

life style[tiab] OR

lifestyle[tiab] OR

life style[mh:noexp])
